# Fractures in professional footballers: 7-years data from 106 team seasons in the Middle East

**DOI:** 10.5114/biolsport.2023.125588

**Published:** 2023-03-08

**Authors:** Aston Seng Huey Ngai, Ian Beasley, Olivier Materne, Abdulaziz Farooq, Montassar Tabben, Souhail Chebbi, Zied Ellouze, Javier Arnáiz, Khalid Alkhelaifi, Roald Bahr, Karim Chamari

**Affiliations:** 1 Aspetar, Orthopaedic and Sports Medicine Hospital, FIFA Medical Centre of Excellence, Doha, Qatar; 2 Manchester City Football Club Ltd, Global Performance Unit, Manchester, United Kingdom; 3 Rangers Football Club, Glasgow, United Kingdom; 4 Oslo Sports Trauma Research Center, Norwegian School of Sport Sciences, Oslo, Norway

**Keywords:** Metatarsal fracture, Leg fracture, Facial fracture, Multiple surgery, Non-union, Stress fracture

## Abstract

Epidemiological studies on fractures in European professional football (soccer) are in abundance. However, such data are lacking in Middle Eastern professional footballers and information on fracture treatment is scarce. The aim of this study is to describe the epidemiology of fractures across seven seasons in Qatar Stars League (QSL) footballers. A prospective study of fractures in professional male footballers over 7 consecutive seasons (2013 to 2020), involving 3255 players and 106 team’ seasons. Time loss and injuries and illnesses were recorded using standardised digital tools in accordance with international consensus procedures. Fractures were recorded according to onset mechanism, location, diagnoses, treatment and return to play. A total of 108 players sustained fractures during 638,247 hours of player exposure (88.9% training and 11.1% matches), representing 2.7% of all time-loss injuries. The incidence was 0.17 fractures per 1000 h of exposure (match and training incidence of 0.9 and 0.07 fractures / 1000 h, respectively), equivalent to an average of one fracture per team per season. Fractures mostly occurred in the feet (28.2%), hands (21.1%), shoulders (11.3%) and head (i.e., face) (9.9%). Mean (median) absence was 71 (47 days), with 4.6% refractures. Only 34.3% of the fractures required surgery and nearly all players (98.1%) returned to play at the professional level. Almost all professional football players with fractures return to play at the same competitive level after an average of 10 weeks of absence (mean absence was 71 ± 81 (median: 47, Inter Quartile Range [14–93]) days). One in ten continue to play with symptoms and one in twenty may refracture. Long-term effects of fractures are still unknown.

## INTRODUCTION

Fractures are not so common in football, with an incidence of just 1 to 2 per season per professional male football team [1]. However, fractures have been shown to be responsible for up to 17% of severe injuries (i.e., time loss > 28 days) [2, 3]. In the UEFA Champions League (UCL), each fracture resulted in a median absence of 32 days (range: 1–278 days), although stress fractures were found to need twice the time to heal (median: 65 days, range: 6–168 days) of sudden onset (traumatic) fractures ((30 days (range: 1–278 days)) [1]. In the same study, fractures occurred mostly due to contact, especially during match play and were located predominantly in the lower limb. In the professional footballers from the Qatar Stars League (QSL), the incidence of fractures in the 2008–2009 season was similar to the UCL findings [4]. However, this study was limited to one season. Neither of the latter studies provided information on treatment [1, 4]. When faced with fractures, clinicians will need to be able to reference clinical service outcomes and return to play estimates. This would allow a reasonable approximation of return to play and enable accurate information for coaching and management staff. It would also bring an awareness of these, more serious injuries, which could potentially be career threatening.

Most previous epidemiological studies on fractures in professional footballers have been done in European clubs and national leagues in temperate environments [1, 5, 6]. Different environmental conditions, variations in training load and/or a different playing style from the European leagues may affect the incidence and prevalence of fractures [7–10]. Therefore, a prospective systematic data collection over multiple seasons would provide accurate information on the morbidity and outcome of fractures in professional footballers in the Middle Eastern region. The aim of the present study was thus to report data on fractures in male professional football players from the QSL over multiple seasons, describing subsequent treatment and return to play timing information.

## MATERIALS AND METHODS

### Study Design

This prospective epidemiological study included fractures in professional male QSL players aged 18 years and above, from up to 17 teams from 2013–2014 through the 2019–2020 seasons. The inclusion criteria were fracture(s) sustained during training or matches in players, playing and/or training with the first-team squad.

### Data Collection

The medical staff (team physician or physiotherapist) of each participating team recorded injury and illness data using an electronic database (the Aspetar Injury and Illness Surveillance Program, using existing surveillance protocols [11, 12]. All injuries, including fractures, were classified using The Orchard Sports Injury Classification System (OSICS, 2017–2020) or previous 2013 to 2016 injuries were recorded with the Sports Medicine Diagnosis Coding System (SMDCS) [12]. These were thereafter independently recoded based on OSICS by two sports physicians. In the rare cases of conflicting outcomes, a consensus meeting was organized to result in the best diagnosis based on the available information and detailed discussion. Details on onset of injury (gradual or sudden, contact or non-contact), location, diagnoses (fracture type), treatment (surgery or conservative), and timing of return to play (RTP) were aligned with the IOC consensus (STROBIS-SIIS) [11].

Injuries diagnosed as fracture(s) were verified by radiological imaging (e.g., X-ray, CT scan, MRI, and/or ultrasound scan) and reported by consultant radiologists. Team physician, physiotherapist and orthopaedic surgeon notes were checked to verify the type and nature of conservative treatment or surgery, date of surgery, medical discharge (return to training with the team) and return to play. Eventual residual symptoms upon discharge, post-surgical complications and/or refractures were also recorded. All efforts were made to retrieve complete data. Data which could not be found or identified were classified as missing. The study is based on a large football epidemiological prospective study approved by the Anti-Doping Laboratory Qatar Ethics Institutional review board (IRB E2017000252).

### Definitions

A fracture was defined as a complete or incomplete discontinuity of bone caused by a direct or indirect force [13]. Injury burden is the product of injury incidence and mean severity, and it is expressed in days lost per 1000 h of player’ exposure [11]. Time loss was calculated from the date of the fracture to the date of return to full participation and availability for match selection [11]. Severity was defined according to the duration of time loss from football according to Fuller et al. [14]. Index injury is the first fracture occurring to one bone recorded during the study. Refractures are defined as a fracture to the same bone and location within one year of the index injury [11].

### Statistical Analysis

Descriptive data are presented as the means, with standard deviation. Median and interquartile range (IQR) are used for fracture severity. The number of time-loss fractures per 1000 player hours of exposure are used for fracture incidence. Independent sample t-test or equivalence nonparametric test were used to compare time loss between binary categorical variables and mechanism of fracture variables. Chi-square test of independence was used to compare the severity percentage according to the mechanism of fracture variables. The level of significance was set at p < 0.05. Statistical analyses was performed SPSS V22.0.

## RESULTS

### Population

For the demographic data of the participants, see Table 1. In total, 3,255 male footballers from up to 17 teams per season (mean ± SD: 15.1 ± 1.6; 106 team seasons) were included from seven consecutive seasons (2013–2014 to 2019–2020) (Table 2). A total of 638,247 h of exposure was recorded. This was equivalent to 6,021 ± 1,235 h per team per season and 196 ± 40 h of exposure per player per season. The training-to-match ratio was 8.0.

**TABLE 1:** Demographic Data of Footballers (mean ± SD).

Demographic Data	Mean ± SD
Age	26.4 ± 4.7
Weight (kg)	70.5 ± 23.9
Height (cm)	168.9 ± 29.1
Total Players	3255
Total Team Seasons	106
Number of players per season	465 ± 46.8
Number of teams per season	15.1 ± 1.6

**TABLE 2:** Incidence and burden of fractures by season from 2013 to 2020. Incidence unit: injury/1000-h of exposure. Burden unit: days of absence/1000-h.

Seasons	Time loss	Time loss injuries	Football Exposure	Incidence rate	95% Confidence Interval		Burden	95% Confidence Interval	
	(Days)	(N)	(Hours)		Lower Bound	Upper Bound		Lower Bound	Upper Bound
2013–2014	1406	17	106160.1	0.2	0.1	0.3	13.2	12.6	14.0
2014–2015	1208	21	99549.3	0.2	0.1	0.3	12.1	11.5	12.8
2015–2016	1552	16	61838.0	0.3	0.1	0.4	25.1	23.9	26.4
2016–2017	980	10	86303.8	0.1	0.1	0.2	11.4	10.7	12.1
2017–2018	866	15	103635.8	0.1	0.1	0.2	8.4	7.8	8.9
2018–2019	967	16	96741.1	0.2	0.1	0.3	10.0	9.4	10.6
2019–2020	659	13	84019.3	0.2	0.1	0.3	7.8	7.3	8.5

### Fracture Incidence and Prevalence

Out of the 3,986 recorded injuries, 108 players (2.7%) sustained a fracture(s). Twelve players (11%) sustained more than one fracture in the same incident ((10 players (9%) had two fractures (e.g., tibia and fibula), and two players (2%) had three fractures (e.g., 2nd, 3rd, and 4th metacarpal fracture)). Incidence and burden of fracture by season from 2013 to 2020 is shown in Table 2.

### Fracture patterns

**FIG. 1:**
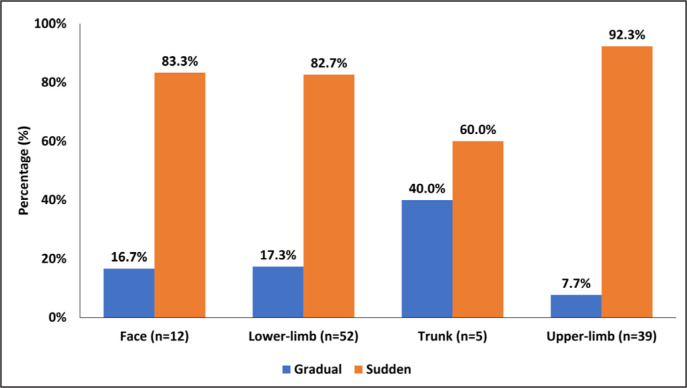
Percentage of sudden and gradual onset fractures according to main body part (N = 108).

**FIG. 2:**
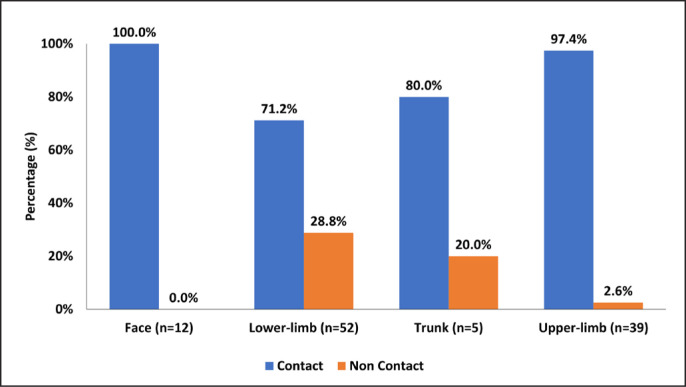
Percentage of contact and non-contact related fractures according to main body part (N = 108).

The fracture patterns according to location is shown in Figures 1 and Figure 2. Sudden onset fractures were more likely to result from a contact ((n = 83, 90.2%; (p < 0.001) than non-contact (n = 9, 9.8%)).. However, there were more non-contact injuries during training (30%) compared to match play (6.3%) (p = 0.001).

### Fractures in Match and training

Match play yielded 12.8 times more fractures than training (incidence: 0.9 vs 0.07 fractures/1000 h). Trunk fractures (n = 4, 80%), and head, neck and face fractures (n = 9, 75%) were more likely to occur during matches than training compared to upper limb fractures (n = 16, 43%) and lower limb fractures (n = 20, 40%). Most of the upper limb fractures recorded from training occurred in goalkeepers (n = 8; 50%).

### Fracture location

The proportion of fractures according to the body location is shown in Table 4. Goalkeepers were 3 times more likely to have upper limb fractures (n = 12) than lower limb fractures (n = 4). Meanwhile, outfield players were almost twice as likely to have lower limb fractures (n = 48) compared to upper limb fractures (n = 27). The proportion of fractures was 59.2% in the dominant lower limb and 38.5% in the dominant upper limb compared to the contralateral side, but there was no statistical significance in comparison with the non-dominant limb (p = 0.068).

### Time Loss

Time loss was 71 (± 81; 47 days), with 63% (n = 68) resulting in severe absence (≥ 28 days). Time loss and severity of fracture according to fracture type, onset of injury, mechanism, and player position are shown in Table 3. There was no significant difference in time loss in relation to the onset of fracture, origin, mechanism, or fracture type.

**TABLE 3:** Time loss (mean ± SD, median and Inter Quartile Range) and severity of fracture (%) according to fracture type, onset of injury, mechanism, and player position.

	Total	Mean ± SD	Median (Percentile 25–75)	Minor (≤ 7 days)	Major (8–28 days)	Severe (> 28 days)
	N (%)	Days	Days	N (%)	N (%)	N (%)
**Origin**						
*Match*	66 (61.1%)	77.0 ± 85.1	47 (14–124)	10 (15.2%)	14 (21.2%)	42 (63.6%)
*Training*	42 (38.9%)	60.9 ± 75.0	47 (16–78)	8 (19.0%)	8 (19.0%)	26 (61.9%)

**Type**						
*Non-Stress Fracture*	97 (89.8%)	70.2 ± 84.6	46 (14–93)	17 (17.5%)	22 (22.7%)	58 (59.8%)
*Stress fracture*	11 (10.2%)	75.6 ± 44.5	68 (47–86)	1 (9.1%)	0 (0%)	10 (90.9%)

**Mechanism**						
*Contact*	91 (84.3%)	66.2 ± 79.3	45 (14–92)	16 (17.6%)	21 (23.1%)	54 (59.3%)
*Non-Contact*	17 (15.7%)	94.9 ± 90.1	78 (47–93)	2 (11.8%)	1 (5.9%)	14 (82.4%)

**Onset of injury**						
*Gradual*	16 (14.8%)	44.1 ± 31.5	44.5 (10.5–74.5)	3 (18.8%)	2 (12.5%)	11 (68.8%)

*Sudden*	92 (85.2%) *	75.4 ± 86.4	47 (14.5–104)	15 (16.3%)	20 (21.7%)	57 (62.0%)
**Player position**						
*Outfield player*	92 (85.2%) *	75.5 ± 84.7	47.5 (15.5–93.5)	12 (13.0%)	19 (20.7%)	61 (66.3%) *
*Goalkeeper*	16 (14.8%)	43.2 ± 51.7	17.5 (4–59.5)	6 (37.5%)	3 (18.8%)	7 (43.8%)

* Significantly different between groups (onset of injury and player position) P < .05

The large majority, 98.1% (106 players) successfully returned to play at the previous level (QSL). Two players retired (1.9%), one unrelated to his fracture.

### Refractures

Refractures was 4.6% (n = 5) with the majority occurring in the lower limb (80%). Of those with refractures, four players had one recurrence and one player had two recurrences (malleolar – supra-syndesmotic). Refractures was in 7.7% of the lower-limb, 2.6% of the upper-limb and none for head-face and neck, or trunk. Most players (60%) who re-fractured had been treated surgically (n = 3, i.e., distal fibula, 5^th^ metatarsal, malleolar (suprasyndesmotic), while 40% had been conservatively managed. (n = 2, i.e., tibial diaphysis, proximal humerus). The mean time to refractures was 231 days (range from 132 to 355 days).

### Treatment

The treatment provided and time loss according to fracture location are shown in Table 4. Hardware placement was used for 81% (n = 30) and closed reduction under anesthesia in 19% (n = 7). Thirteen players (43%) needed more than one surgery, for hardware removal. Lower-limb fractures were more often treated with multiple surgeries (two or more) (47.4%) than upper-limb fractures (16.7%). None of the older players (> 28 years) who sustained fractures during training needed surgery. Only one goalkeeper needed surgery for an upper limb fracture (n = 1, 9.1%).

**TABLE 4:** Time loss (mean, SD and Median) according to treatment and fracture location.

	No Surgery N = 71 (65.7%)			Surgery N = 37 (34.3%)		
	Total	Mean ± SD	Median (Percentile 25–75)	Total	Mean ± SD	Median (Percentile 25–75)
	N (%)	Days	Days	N (%)	Days	Days
**Main body part**						
*Head-face-Neck*	7 (9.9%)	12 ± 8	14	5 (13.5%)	29 ± 25	20
Lower limb*	32 (45.1%)	56 ± 37	51	20 (54.1%)	185 ± 106	156
*Trunk*	5 (7.0%)	42 ± 33	32	0	—	—
*Upper limb*	27 (38.0%)	29 ± 36	14	12 (32.4%)	78 ± 73	57

**Location**						
*Ankle*	3 (4.2%)	21 ± 19	16	2 (5.4%)	128 ± 45	128
*Chest/Ribs/Upper Bac*k	3 (4.2%)	19 ± 13	19	0	—	—
Foot*	20 (28.2%)	57 ± 43	52	6 (16.2%)	113 ± 36	105
*Forearm*	1 (1.4%)	59	59	1 (2.7%)	42	42
Hand*	15 (21.1%)	14 ± 18	6	6 (16.2%)	60 ± 58	42
*Head*	7 (9.9%)	12 ± 8	14	5 (13.5%)	29 ± 25	20
*Knee*	4 (5.6%)	62 ± 23	57	1 (2.7%)	219	219
Lower Leg*	5 (7.0%)	62 ± 22	51	10 (27.0%)	251 ± 107	248
*Pelvis/Low Back*	2 (2.8%)	75 ± 16	75	0	—	—
*Shoulder*	8 (11.3%)	55 ± 52	46	4 (10.8%)	49 ± 34	59
*Thigh*	1 (1.4%)	53	53	0	—	—
*Wrist*	2 (2.8%)	28 ± 25	28	2 (5.4%)	188 ± 99	188

* Significantly higher days loss in surgery compared to no surgery, P < 0.05

There was no significant difference in the number of surgeries done for gradual onset (stress) fractures (45.5%) compared to sudden onset (traumatic) fractures (32%) (p = 0.20). All stress fractures requiring surgery were located at the 5^th^ metatarsal bone with four (80%) requiring only one surgery and the remaining one (20%) requiring two surgeries.

Complications were seen only in surgically treated fractures (22%, n = 8). These comprised: non-union (n = 2), stress fracture (n = 2), delayed union (n = 1), osteoarthritis (n = 1), deformity (n = 1) and infection (n = 1). The lower limb was the main location for complications (75%) followed by the upper limb and face, with 12.5% each. Only 9.3% (n = 10) had residual symptoms at return to play, i.e., pain (7, 6.5%), swelling, instability and restricted movement (1 each).

## DISCUSSION

This study showed that fractures in QSL male professional footballers occurred mostly by sudden onset and contact mechanism; furthermore, fractures were located mostly in the lower limbs and occurred during match play rather than training. Lower limb fractures were also more likely to require surgery; naturally, these needed extended time loss, unlike upper limb fractures.

Fractures representing 2.7% of total time-loss injuries sustained by players was in the range from 2.6% to 4% of previous studies from European professional leagues [3, 15, 16].

Fracture incidence (0.17 fractures per 1000 h of exposure) was similar to the 0.19/1000-h found in German Bundesliga but lower than the 0.27/1000-h from the UCL study [1, 6]. We showed that there was an average incidence of 1.0 fracture per team per season in the QSL. The proportion of stress fractures (10.2%) did not substantially differ from earlier studies, between 4.5% to 11.3% [1].

Slightly higher than the 75% from the UCL study, 85% of the fractures occurred with sudden onset, due to contact, and during match play [1]. The incidence of sudden onset (previously reported as traumatic fracture) and stress fractures (also described as overuse) was similar to the UCL and Swedish Super league findings [1, 5]. Unlike previous studies, the higher proportion of injuries in the dominant limb as seen for soft tissues (predominantly muscle and ligament injuries) was not observed for fractures in our study [15].

In this study, lower limb fractures (48%) were the most common, in accordance with most European professional league study findings (35.3% to 50%) [1, 3, 6]. It has been suggested that the use of shin guards prevented some leg fractures, based on a previous laboratory study, and obviously the use of these is mandatory in the QSL as in other leagues [17]. However, there is no mandate on the type or size of shin guards rendering efficiency potentially inconsistent from the size perspective. Leg fractures have been shown to cause absences of 251 ± 107 days (around 36 weeks (about 8 and a half months), comparable to the time loss after ACL surgery [18]. In practical terms, this amounts to the loss of almost a full season in the major international leagues (usually a player season including pre-season is around 48 weeks (about 11 months), and therefore, any means of eventually preventing such events would have a significant impact on football players careers.

Upper limb fractures (36%) in our study were in the upper range compared with the literature, with the Swedish Premier League (28.6%) and the German Bundesliga (24.9%) situated in a middle-range while the UCL (14%) presented a lower figure [1, 3, 6]. In the goalkeepers of our study, upper limb fractures occurred more commonly during training possibly due to higher frequency of dives, jumps, high speed change of direction compared to matches, as suggested in a review [19]. Interestingly, AlAttar et al. recently suggested that FIFA 11+S upper limb exercises were beneficial to amateur footballers in reducing upper limb injuries of moderate severity [20]. Goalkeeper training increases their ability to land safely and anticipate player or ground contact [19]. Hence, these factors may be part of the reason why the more serious upper limb fractures, i.e., those requiring surgery, were less common.

Head, face, and neck fractures (all facial in our study) (11%) were less common than in European leagues ranging from 16.8% to 30.3% [1, 3, 6], possibly due to fewer headers and different playing styles in players in the QSL, although this has not been objectively assessed to date. As suggested in previous studies, stricter game rules, referee implementation and avoidance strategies in tackling, heading and ball control, in addition to a potential lower engagement in duels (especially aerial ones) may also have contributed to fewer facial fractures in our study [21, 22]. Time loss due to these fractures was similar to findings in European leagues. Indeed, the players of our study were allowed early return to play after facial fractures thanks to thermoplastic face masks as consistently observed in major international leagues during the last decade [1, 6].

In an earlier study of recreational footballers, only 20% of fractures needed surgery but in our study with professional footballers, the proportion was greater (34.3%) [23]. Previous studies have suggested that athletes who are surgically treated with post-traumatic metacarpal, clavicle, tibial and fibular, or fifth metatarsal fractures have fewer complications, and a quicker return to play, but in our study, this was seen only with fifth metatarsal fractures [24–26]. We showed that time loss due to post-traumatic tibia and fibular fractures was similar to the European leagues and American Football findings [27, 28].

Refractures was slightly less common in the QSL (4.6%) compared with the UCL (7.7%) [1]. This was mainly due to a much lower refractures in the upper limb (2.6% vs 14.3%), trunk (0% vs 10%) in the QSL than in UCL [1]. We speculate that the longer absence to treat these fractures may have contributed to the observed much lower refractures in QSL as the longer time allows for improved fracture consolidation [29]. However, the lower limb refractures (7.7%) in QSL was similar to the UCL [1].

### Return to play

Despite a high percentage of players (63%) sustaining fractures causing severe absence (longer than 28 days), only two (1.9%) out of 108 players retired. Therefore, as with earlier studies, almost all professional players (98.1%) returned to play at the previous level, i.e., same level of the competition [23]. One player left the league after a prolonged absence of 403 days, 3 surgeries, and non-union, whereas the other left to play football abroad. Of the 9 players who had post-surgical complications, 8 (88.9%) returned to play whereas the remaining player retired upon discharge from treatment. The residual symptoms seen upon return to play in 10 players (9.3% of 108) did not affect their ability to train and play competitive matches. The high motivation, level of responsibility and financial needs in professional footballers and/or the competency of the team medical staff (including subspeciality musculoskeletal radiologists, orthopaedic surgeons, sports medicine physicians, physiotherapists and biomechanists) supporting them may have contributed to their ability to continue playing despite having symptoms [30].

### Strengths and limitations

The study was done with systematic recording and verification of injury data of professional male footballers and importantly, included data on fracture treatment. However, female, and amateur players were not included and obviously deserve specific dedicated studies. There were some missing data about training and match exposure, mechanism of injury and time of injury (minutes in the match or training session). It was difficult to ascertain the nature of contact or the inciting event and identify any referee sanction without video analysis [21]. We also could not utilise the global positioning system technology (GPS) data to quantify training and match load, although a previous author suggested that playing intensity and injury may be correlated [31]. Such data could have been especially useful to determine the potential effect of external load in the contribution of lower limb stress fractures risk during match or training [32, 33].

The choice of treatment depends on multiple factors which may affect time loss e.g., (i) shared decision making between the player, physician, surgeon and/or coach, (ii) risk taking behaviour, (iii) period of season with the importance of upcoming matches potentially influencing the decision (iv) site and classification of fracture and/or (v) player position and importance in terms of being ranked by the coach as first, second or even third in his specific field position [34–38]. The rehabilitation programme differed with fracture location, type of fracture, length of immobilisation and whether the fracture was treated conservatively or surgically. In previous studies, focal extracorporeal shockwave therapy has shown a promising response on non-union and delayed union fractures. However, their effect on return to play has not been studied in athletes [39]. The 12-month follow-up of refractures adopted in our study prevented the possibility of missing some refractures, as previous studies have shown that refracture may occur up to 10 months from the first fracture [28, 40]. But with the one-year post-fracture limit, we might have missed other long-delay refractures, even though actual consensus is setting the limit to one year. Our data did not include a 5-year follow-up for each fractured player (whenever possible, in terms of player’ age and availability) which could have been made to study player retention and long-term effects of the fracture on the player’s career as suggested in Lavoie-Gagne et al’s study [28].

## CONCLUSIONS

Almost all professional football players who sustained fractures returned to play at the previous competitive level after an average of 10 weeks, although leg fractures resulted in up to 27 weeks of absence. One case in twenty suffered a refracture requiring further treatment, whilst one-tenth of the injured players continued to play despite having mild symptoms. The long-term effects of football fractures are still unknown and warrants investigation.

## Data sharing

The datasets used and/or analysed during the current study are available from the corresponding author on request.

## Patient and Public Involvement

Patients or the public WERE NOT involved in the design, or conduct, or reporting, or dissemination plans of our research.
